# Threat and Risk Analysis-Based Neural Network for a Chemical Explosion (TRANCE) Model to Predict Hazards in Petroleum Refinery

**DOI:** 10.3390/toxics11040350

**Published:** 2023-04-07

**Authors:** Lalit Rajaramji Gabhane, NagamalleswaraRao Kanidarapu

**Affiliations:** School of Chemical Engineering, Vellore Institute of Technology, Vellore 632014, Tamil Nadu, India

**Keywords:** process reliability, refinery, risk analysis, TRANCE model

## Abstract

Risk analysis and prediction is a primary monitoring strategy to identify abnormal events occurring in chemical processes. The accidental release of toxic gases may result in severe problems for people and the environment. Risk analysis of hazardous chemicals using consequence modeling is essential to improve the process reliability and safety of the refineries. In petroleum refineries: toluene, hydrogen, isooctane, kerosene, methanol, and naphtha are key process plants with toxic and flammable chemicals. The major process plants considered for risk assessment in the refinery are the gasoline hydrotreatment unit, crude distillation, aromatic recovery, continuous catalytic reformer, methyl–tert–butyl–ether, and kerosene merox units. Additionally, we propose a threat and risk analysis neural network for the chemical explosion (TRANCE) model for refinery incident scenarios. Significantly, 160 attributes were collected for the modeling on the basis of the significance of failure and hazardous chemical leaks in the refinery. Hazard analysis shows that the leakages of hydrogen and gasoline at the gasoline hydrotreatment unit, kerosene at the kerosene merox plant, and crude oil at crude-distillation units were areas of profound concern. The developed TRANCE model predicted the chemical explosion distance with an *R*^2^ accuracy value of 0.9994 and *MSE* of 679.5343.

## 1. Introduction

Rapid industrialization and the use of more advanced technology, materials, plants, and machinery have led to a significant jump in petroleum product consumption [1]. A petroleum refinery regularly handles many flammable and explosive products in large volumes in processing units and storage tanks. It is prone to major accidents such as fire, explosions, and the dispersion of toxic and flammable substances [2,3] due to the handling, processing, and storage of highly flammable substances and the operation of the facilities at elevated temperatures and pressure or cryogenic conditions [4]. These potential hazards become aggravated due to processing upsets, extreme physical conditions, and the accidental release of flammable hydrocarbons, causing major incidents [5]. The statistical data show that the frequency of fire and explosive hazards in petroleum refineries is increasing [6]. Equipment failure and the lack of a safety culture are the primary reasons for incidents. A catastrophic incident also leads to fire and explosions [7]. In a refinery complex, the gasoline hydrotreater unit (GTU), continuous catalytic reforming unit (CCR), methyl–tertiary–butyl–ether unit (MTBE), crude oil distillation unit (CDU), kerosene merox unit (KMU), and aromatic recovery unit (ARU) are the central functional units causing fire and explosive hazards [8]. Petroleum refineries have good safety regulations, standards, codes of practice, checklists, and operational protocols that provide multiple levels of protection against incidents [9]. However, despite that, the industry is still vulnerable to significant accidents such as fires, explosions, and the dispersion of toxic and flammable substances [10,11]. The Indian Oil Corporation Vadodara accident revealed the need to clearly understand the various types of accidents in oil installations and the importance of emergency operation centers [12]. There have been several global incidents of fires and explosions in the oil and gas industry, and there are various case studies and reports on accidents in this industry [13,14]. Emergencies arise due to causes such as explosions in a processing plant, accidents in storage facilities, during transportation, or due to technological failures, inappropriate or inadequate plant safety design, arson, malicious acts, human error, and natural calamities. Therefore, it is essential to investigate petroleum refineries’ worst possible safety hazards, evaluate their impact on people, property, plants, and society, and devise risk-control strategies [15].

Quantitative risk assessment in the processing industry is vital to protect workers and the environment [16]. Consequence analysis is a part of risk assessment, and its qualitative and quantitative methodologies help in estimating significant losses in the processing industry [17]. The accidental release of toxic gases, fire, explosions, and the dispersion of combustion products are some hazardous scenarios in processing industries [18]. Each scenario and its consequences could be calculated using modeling software such as CFD, ALOHA [19], WISER, and PHAST. Thermal radiation models estimate the amount of released heat due to explosions [20]. The results of consequence analysis are used to prepare emergency response plans [21]. Vapor cloud explosions in refineries and LPG plants are more severe in low-wind conditions as the vapor travels due to gravity [22].

Historical data availability is influenced by developing data-driven techniques in processing safety from fault identification to risk assessment. Past risk-assessment methods depended on steady-state statistical data to predict failure occurrence, causes, and consequences in the processing industry [23].

ML methods in risk assessment from corrosion cracking are applied with big data to integrate the Internet of Things [24]. Several soft-computing techniques and their application in safety evaluation were suggested for strategic decision-making in hazard management [25].

The critical decision-making parameter is effective for risk analysis measures regarding the worst-case chemical explosion failure in processing industries. Multimodal failure in industrial processes is difficult with a single solution at steady-state conditions. Therefore, several local data-driven strategies with different algorithms for the dynamic failure of processing components are used in pipeline incidents [26]. A multimodal machine-learning framework for process modeling with feature selection was implemented in the distillation column and achieved acceptable results. Potential hazardous chemicals in drinking water were predicted with two different supervised and unsupervised ML models. The prediction results were compared and had less than 5% false detection [27]. A multilayer perceptron neural network was developed with an accuracy of 84% for historical oil and gas pipeline data [28]. A comprehensive review of machine-learning approaches for oil and gas pipeline failure predictions was also conducted [29].

A new ANN framework, the operable adaptive sparse identification of systems (OASIS), was developed for chemical processing and successfully applied to reactor-separation risk analysis [30]. Risk assessment using the OASIS of an oil and refining plant was conducted from incidents on the basis of atmospheric and internal conditions and fault prediction.

An ANN framework using a Bayesian algorithm was proposed to predict faults of and assess the risk from corroded pipeline incidents [31]. Predictive analysis was performed on the LNG bunker price using a recurrent neural network, long short-term memory (LSTM), and gated recurrent methods. LSTM best suits highly volatile data in fault forecasting [32].

The literature shows that fire and explosion analyses were performed for storage tanks but not other critical process units of the refinery. In this context, the authors were motivated to conduct the present research on fire and explosion hazard analysis for the essential units of the refinery. The present study addresses the gap and focuses on analyzing worst-case fire and explosion scenarios in a petroleum refinery, which may occur during its operational life due to mechanical failures, sabotage, or natural calamities.

The present study aims to carry out the fire and explosion hazard analysis for critical plants of the refinery and to estimate the probable fire and explosion threats that will seriously impact people in the refinery, plant, equipment, and the surrounding community. The critical plants considered for the analysis are the gasoline hydrotreater unit (GTU), continuous catalytic reforming unit (CCR), methyl–tertiary–butyl–ether unit (MTBE), crude oil distillation unit (CDU), aromatic recovery unit (ARU), and kerosene merox unit (KMU). Additionally, different failure scenarios in the refinery plant and the measured threat zones (probably red, orange, and yellow) based on the distance traveled by the explosion of hazardous chemicals data were collected from NSCI (the National Safety Council India). The influenced parameters (wind speed, air temperature, and relative humidity) are considered to spread the exploded chemicals. This work aims to develop a TRANCE model for predicting threat- and risk-zone (red, orange, and yellow) distance with real-time industrial data when multimode failures occur at different process units in a refinery plant.

## 2. Materials and Methods

Industrial data from a large petroleum refinery complex in western India is used for the study. The data include process flow diagrams, process information, and regional meteorological data on the refinery’s location. The refinery is on around 500 acres of land surrounded by industrial estates, residential colonies, and other commercial establishments. This refinery has a present refining capacity of about 12 MMTPA. The main products produced in the refinery are naphtha, motor spirit, hexane, benzene, toluene, mineral turpentine oil, kerosene, jet fuel, diesel, light diesel oil, fuel oil, LPG, and bitumen. A systematic hazard-identification technique HAZID was followed in this study. The entire plants and pipelines in the refinery under this study are divided into several isolated sections. Each section was examined to identify the likely failure scenario regarding the leak size, the quantity of hazardous material contained in the isolatable section, and the estimated duration of the leak. Sixteen potential worst-case scenarios (seven each in summer daytime and winter night-time) were considered to perform risk analysis with the ALOHA hazard modeling tool and are given in Table 1.

### TRANCE Topology

**Figure 1:**
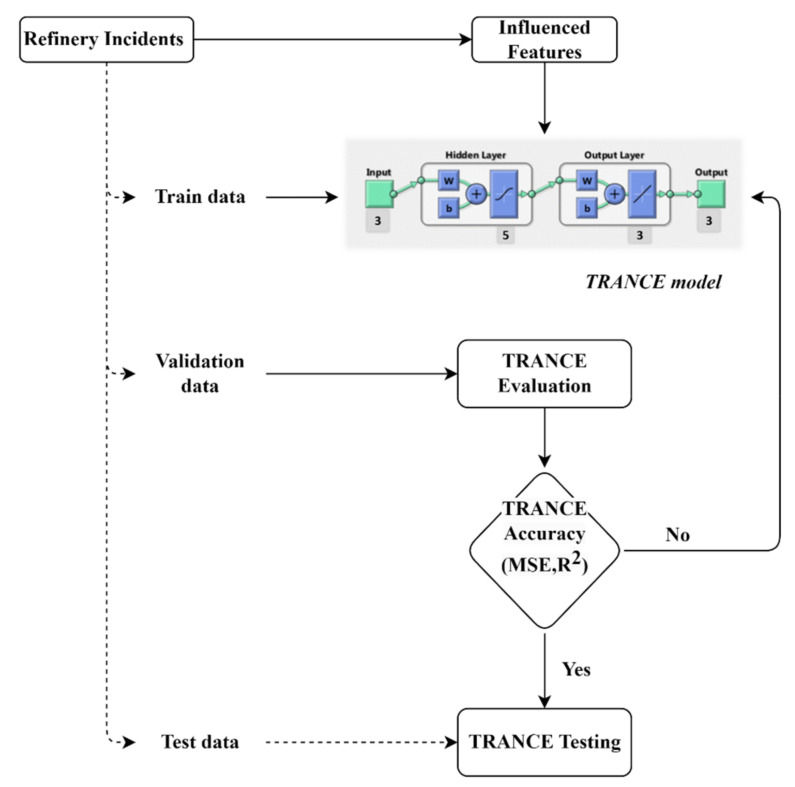
TRANCE framework development methodology.

Soft-computing techniques via ANNs, FL (fuzzy logic), ML (machine learning), PR (probabilistic reasoning), and EA (evolutionary algorithms) have proved incredible efficiency for problem-solving in a variety of applications, specifically in risk assessments to support decision-making processes in an economical and time-saving manner. ANN is the most promising method to build a strong relationship between processes’ input and output features. ANN can be used in numerous applications such as system identification, modeling, process control, fault identification, and risk assessment. The intellectual task performance of ANN is similar to brain neurons by gathering and storing knowledge during the training stage. ANN is noteworthy in developing a surrogate process model of a real one. Perceptron artificial nodes could correlate and create a new response for non-linear and complex process data. A feed-forward multi-layered neural network is preferable for chemical process applications. A threat and risk artificial neural network for chemical explosion (TRANCE) incident structure was developed to transfer signals from input to output features. The performance capability of the TRANCE structure depends on located hidden layers/neurons between these input–output features based on activation and summation functions. The training algorithm and activation function are the key parameters that affect the TRANCE structure performance. The optimized TRANCE structure for refinery incidents is represented in Figure 1. This optimized structure has three input features, a hidden layer with five neurons of the sigmoid activation function, and an output layer with three neurons for three output features of the linear activation function. Levenberg–Marquardt (LM) algorithms are used to train the learning process of TRANCE for minimum error value. LM will develop a pattern by adjusting the weights and biases between input and output features instead of memorizing large datasets. LM is a trainlm back-propagation algorithm, which deals with large datasets with high convergence speed for ANN and optimizes the features iteratively until the predicted values reach an acceptable level. The specifications and overview of the proposed model information are provided in Table 2. The proposed network was applied to predict the safety distance of the refinery dataset generated from ALOHA. We examined the proposed framework capability with multi-failure scenarios over the single fault prediction done earlier. The worst-case scenarios identified from the HAZID and the affected weather conditions were applied to the ALOHA to generate safety distance measures. The effect of chemical explosion distance based on the weather conditions, air temperature, relative humidity, wind direction, wind speed, etc., were considered to measure the vicinity of hazardous chemicals. The chemical distances are classified as red, orange, and yellow zone depending on chemical concentration and the severe risk to humans and/or the environment. One hundred and sixty (160) different weather conditions were applied, and the safety distances were measured. The noise and error in datasets were clustered, and unnecessary data were removed. Datasets were divided for training, validation, and testing purposes as 70%, 15%, and 15% of the total, respectively. The training data were applied to the TRANCE model to train the model and to establish a correlation between the input and target signals. The input layer TRANCE model has three features (air temperature, relative humidity, and wind speed) that are significant effects of a chemical explosion. The output layer of TRANCE has three targets, namely red, orange, and yellow threat zones, for risk assessment. The validation datasets were used to evaluate the trained model and tune the mandatory parameters to attain the high-performance accuracy of the TRANCE model.

## 3. Process Description

**Figure 2:**
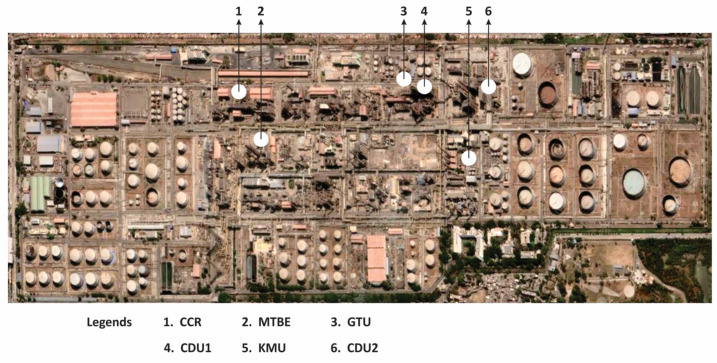
Satellite image of a petroleum refinery.

**Figure 3:**
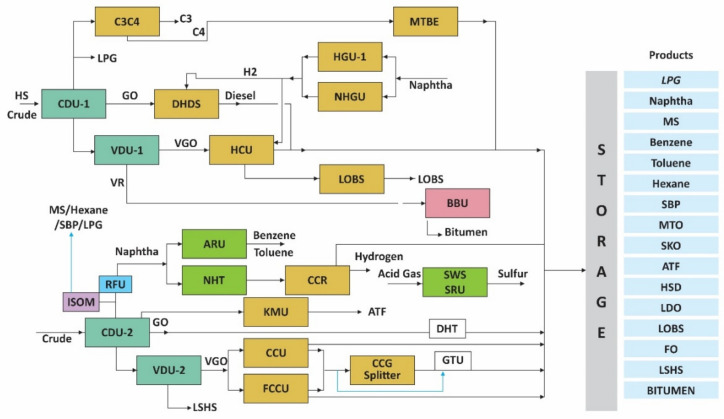
Block flow diagram of refinery processes.

The selected large-petroleum refinery is in western India. The Google map image of the refinery is given in Figure 2. The refinery is situated on around 500 acres of land, surrounded by industrial estates, residential colonies, and other commercial establishments. This refinery has a present refining capacity of around 12 MMTPA. The main products made in the refinery are naphtha, motor spirit, hexane, benzene, toluene, MTO, diesel, light diesel oil, fuel oil, kerosene, jet fuel, LSHS, LPG, and bitumen. The refinery process operations are illustrated in the block flow diagram in Figure 3. The major process plants of the refinery considered for this study are crude-distillation units (CDU-1 and 2), gasoline hydro treatment units (GTU), kerosene (KMU) units, continuous catalytic regeneration unit (CCR), and methyl–tert–butyl–ether (MTBE) unit.

The crude-distillation unit (CDU) is the first plant in the refinery. After desalting, the crude feedstock is preheated using recovered process heat. The feedstock then passes through a direct-fired crude-charge heater and later into the vertical distillation column just above the bottom at a pressure slightly higher than atmospheric pressure and temperatures of about 350–370 °C. Except for the heaviest fractions, all other fractions flash into vapor. As the hot vapor rises through the tower, its temperature decreases. Heavy fuels and asphalt residues are removed from the bottom. The major products, including lubricating oil, heating oil, kerosene, gasoline, and uncondensed gases, are drawn from the successively higher points of the distillation tower.

The topped crude withdrawn from the bottom of the CDU is heated to temperatures ranging from 370 to 420 °C in the vacuum-distillation unit (VDU). The heated topped crude is flashed into a multi-tray vacuum-distillation column which operates at an absolute pressure ranging from 350 to 1400 kg/m^2^. The catalytic-cracking unit (CCU) uses heat, pressure, and catalysts to convert heavy oils into lighter products, such as more valuable gasoline and distillate blending components. Gas oils from atmospheric distillation, vacuum distillation, coking, and de-asphalting processes are generally used as feedstocks. These feedstocks have a boiling point ranging from 340 to 540 °C.

The isomerization unit (ISOM) converts n-butane, n-pentane, and n-hexane into their respective iso-paraffins with extremely high-octane numbers. The isomerization unit is critical while converting the n-butane to isobutane and provides additional feedstock for the alkylation unit. Additionally, to convert the normal pentanes and hexanes into higher-branched isomers for gasoline blending. The isomerization process is similar to catalytic reforming, where-in the hydrocarbon molecules are rearranged. However, unlike catalytic reforming, isomerization only converts the normal paraffin into iso-paraffins. The naphtha hydrotreater unit (NHT) with hydrogen is used to desulfurize the naphtha fraction from the crude oil distillation and other units in the refinery. The gasoline hydrotreater unit (GTU) employs a catalytic chemical process to remove sulfur (S) from gasoline to reduce the sulfur dioxide (SO_2_) emissions that result from using gasoline while maintaining its octane value. GTU uses hydrogen to desulfurize some other distilled fractions removed from the crude oil distillation unit.

The refinery’s kerosene merox unit (KMU) desulfurizes the kerosene and jet fuel by oxidizing undesired mercaptans to organic disulfides. A fluid catalytic-cracking unit called FCCU converts the heavier, higher-boiling fractions from the crude distillation unit to lighter, lower-boiling-point fractions. In the hydrocracker unit (HCU), hydrogen converts heavier fractions from the crude oil and vacuum-distillation units into more lightweight products. The aromatic recovery unit (ARU) removes benzene and toluene from naphtha in the refinery.

## 4. Results and Discussions

Sixteen (eight each in daytime and night-time) hazardous leak scenarios at significant process units in the refinery were modeled to analyze the threat zones. The leakages considered are hydrogen gas at CCR and GTU, gasoline at GTU, methanol at MTBE, crude oil at CDU1, naphtha at CDU2, toluene at ARU, and kerosene leak at the KMU plant. The ALOHA modeling program output results for each leakage scenario for different process plants of the refinery are analyzed. The threat zones are categorized into red, orange, and yellow based on the leaked chemical concentrations. ALOHA’s default toxic level of concern values is considered to decide the type of threat zone in all scenarios. Stability class, wind speed, leak diameter, spill area, chemical release duration, and gas density are significant variables for the threat zones. Among them, the leak diameter plays a crucial role because chemical release quantity is a function of the leak diameter section.

### 4.1. Flammable Area of Vapour Cloud

**Figure 4:**
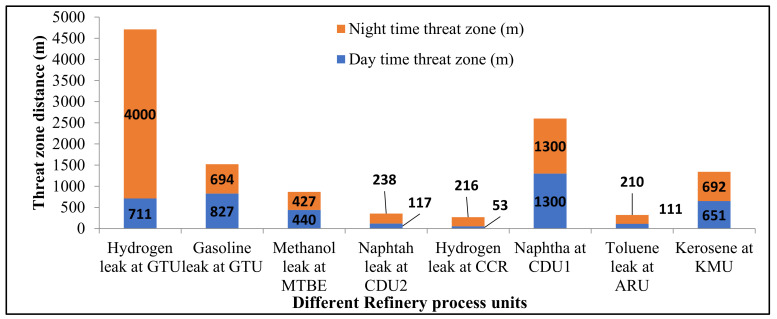
Threat zones for a flammable area of the vapor cloud.

The worst hazard levels due to the formation of a flammable area of vapor cloud during a summer day and winter night-time for process plants were plotted in Figure 4. The vapor cloud’s threat zones extend beyond refinery battery limits. For the hydrogen gas leak at GTU during the winter night, the vapor cloud spread up to 4000 m. The flammable vapor cloud for a gasoline leak at GTU spread up to 827 m, and for a crude oil leak at CDU1 spread to 1300 m during daytime summer. Results indicate that the winter night-time threat zones are slightly more significant than the summer daytime threat zones. Compared to the other process units, the hydrogen leak at GTU spreads more area, and there is a scope for vapor cloud explosion if the escaping gas ignites immediately.

### 4.2. Jet Fire from Thermal Radiation

**Figure 5:**
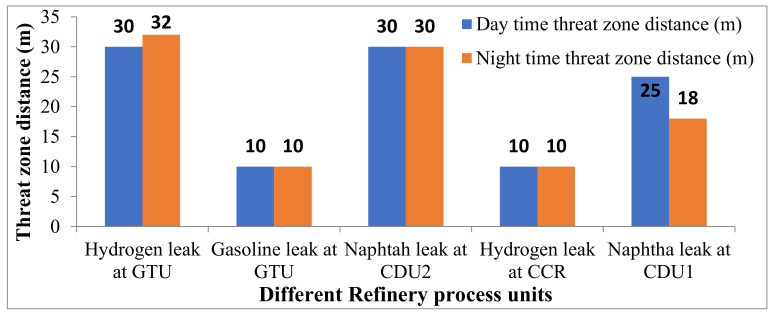
Threat zones for jet fire.

Lethal thermal radiations for 37.5 kW/m^2^ intensity from a jet fire can be observed in Figure 5. The worst-case hazard results for the scenario of full-bore rupture (FBR) indicate that in case of hydrogen leak at the GTU process plant, jet fire from thermal radiation reached a maximum distance of 32 m during the winter night-time. These thermal radiations may cause 100% fatalities, significant damage to the plant, equipment, buildings, and property, and also causes first-degree burns.

### 4.3. Fireball Thermal Radiation

**Figure 6:**
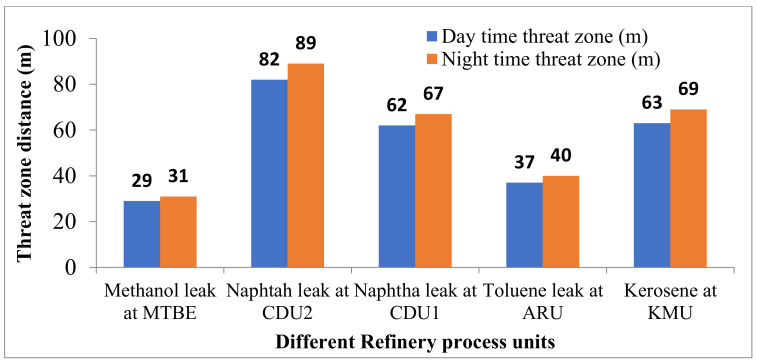
Threat zones for fireball radiations.

Fireballs radiations of 37.5 kW/m^2^ intensity for the process units are provided in Figure 6. This can cause the spread of fire by domino effect to other areas. Figure 6 shows the worst-case hazard zone with a maximum threat-zone distance of 83 m for the naphtha leak at CDU2.

### 4.4. Blast Force from Vapor Cloud Explosion

**Figure 7:**
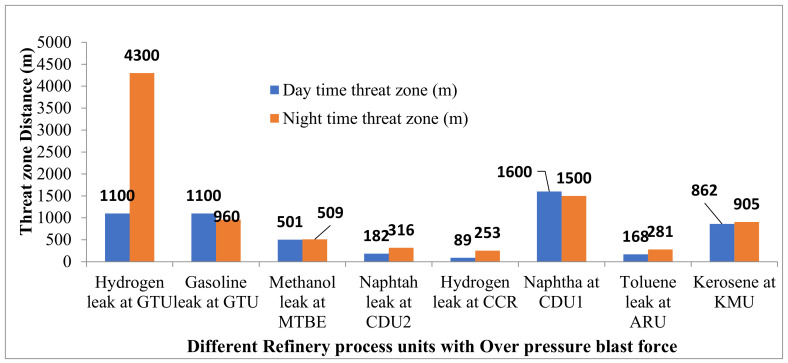
Threat zones for blast force from vapor cloud explosion.

The overpressure blast effect due to the vapor cloud explosion will destroy buildings and cause severe injuries to the people residing at nearby places. Figure 7 shows that the impact of blast force will be severe during the winter night with a pressure of 8 psi magnitude that may reach up to 4300 m causing the destruction of buildings. Severe injuries will be caused to people within a distance of 4300 m in the downwind direction.

Compared to the hazards created by the other process plants, the catastrophic rupture of a large hydrogen pipeline at GTU is the most significant hazard in the refinery. It will significantly impact if the incident occurs during the winter night.

### 4.5. Performance of the TRANCE Model

**Figure 8:**
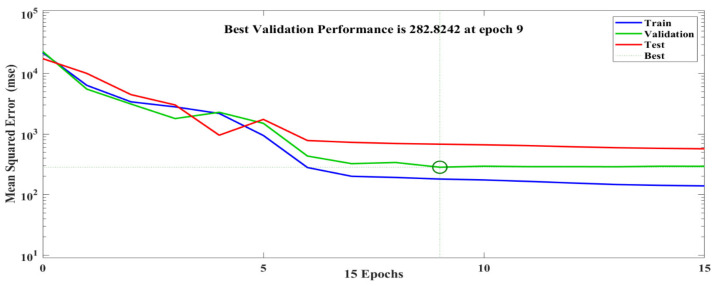
Leaning rate curve for TRANCE model performance with training, validation, and testing datasets.

The separated datasets with 112,24,24 for train, validation, and testing were iterated to achieve the minimum error value of the TRANCE model. In Figure 8, the learning rate of the proposed model was validated with *MSE* values of training, validation, and testing of TRANCE model datasets presented. Train, test, and validation curves show a minimum error of 282.82 at epoch 9 of total epochs 15. The curves have similar behavior from epoch 9 steadily without overfitting or underfitting. Thus, the TRANCE model for risk assessment was established successfully. The performance of the TRANCE model can also be evaluated from another essential parameter called the coefficient of determination (*R*^2^). The mathematical expressions for *MSE* and *R*^2^ are represented as follows [33]:

$$MSE=\frac{1}{N}\overset{N}{\underset{i=1}{\sum }}{\left(y_{TRANCE,i}-y_{experimental,i}\right)}^{2}$$

(1) $$R^{2}=1-\frac{\sum_{i=1}^{N}(y_{TRANCE,i}-y_{experimental,i})}{\sum_{i=1}^{N}(y_{TRANCE,i}-y_{m})}$$

Here, $y_{TRANCE,i}$ represents prediction values from the proposed model, $y_{experimental}$ is true values or experimental target features and $y_{m}$ is the mean of all target values.

**Figure 9:**
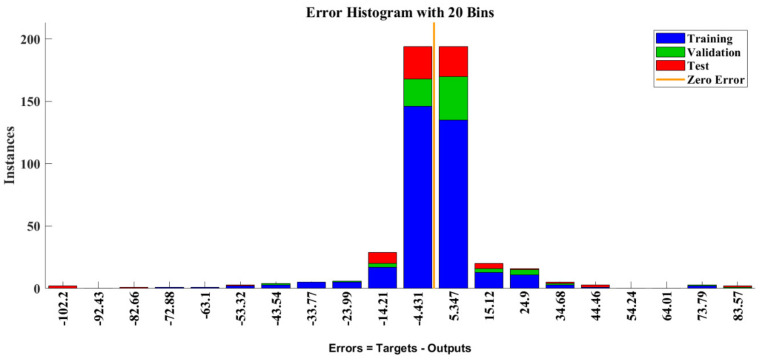
Error histogram plot of training, validation, and testing datasets.

Error values between target and TRANCE outputs are mentioned in Figure 9, with the datasets on the vertical axis and the error on the horizontal axis. The error value displayed a linear change trend in testing, validation, and training datasets. It demonstrates that most of the data’s accumulated peak values are near zero and reveal the superior prediction strength of the TRANCE model.

**Figure 10:**
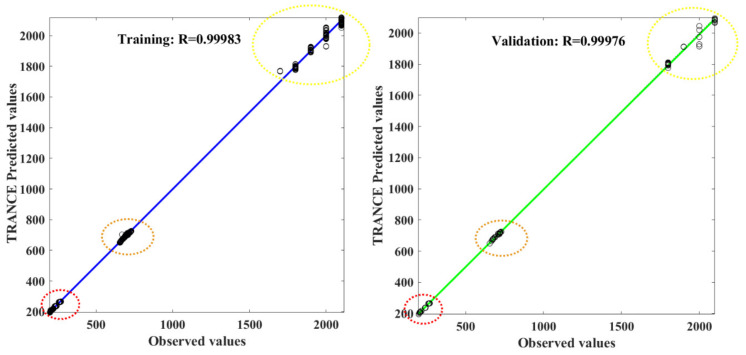
The correlation coefficient for three threat zone prediction values from the TRANCE model in training and validation.

A TRANCE-based model is developed by utilizing preprocessed industrial dataset of refinery plant failure scenarios. The dataset preprocessing is performed using Matlab 2022a with a computational time of 30 min on a 5 Core computer with 8 GB RAM. The data sets were trained and validated through this TRANCE model, and the error values are acceptable. Testing has been done to predict the threat-zone response from the TRANCE model for the new datasets, and the predicted values are collected. The obtained error results in all phases are presented in Table 3. In Figure 10, the accuracy of the TRANCE model in the training and validation phase is quite good, with the coefficient of determination values of 0.9998 and 0.998, and the error values (*MSE*) are 180.70 and 282.82, respectively, which is higher than the SVM-based risk assessment of rock bolts failure in coal mines [34].

**Figure 11:**
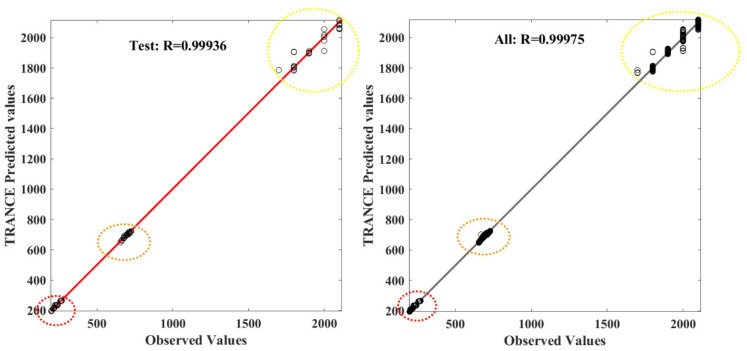
The correlation coefficient for three threat zone prediction values from the TRANCE model in testing and all together.

The overall training, testing, and validation datasets are presented in Figure 11, and three zones are highlighted. This Figure is evidence of the high-precision performance of the TRANCE model in all three output features, orange and yellow zones, which are highlighted with respective colored circles. Similarly, Figure 11 represents the testing ability of the TRANCE model with high precision with the evidence majority of predicted values from all three threat zones lie on a linear regression line with an *R*^2^ value of 0.9994 and *MSE* value of 679.53. Data-driven model-based corrosion risk-assessment prediction from a gradient-boosting algorithm has less efficiency of *R*^2^ value of 0.98 compared with the TRANCE model [35].

### 4.6. Limitations of the TRANCE Model

A large modern refinery in India was considered for the pilot study. The rudimentary refining processes in petroleum refineries in the Indian context are similar. This study focuses on predicting the hazardous-release threat zones in case of release from various process units in the refinery. Therefore, the model will be helpful for other refineries as well. Industrial data from a large petroleum refinery complex in western India were used for the study. The failure scenarios data were used as input for the ALOHA. The TRANCE model dataset is generated from the weather conditions of the location and failure scenarios of the refinery together. In this context, authors can generate datasets by changing weather conditions in ALOHA based on their plant location. The same data can apply to this TRANCE model, but this proposed accuracy may vary. This model can change or modify the hyperparameters such as training algorithm, number of neurons, layer size, epoch size, and dataset splitting to get accurate predictions from the model.

## 5. Conclusions

The consequence modeling of a real-time petroleum refinery was carried out to analyze the fire and explosion hazards present at various key process plants and are considered for the analysis of worst-case fire and explosion scenarios. Hazard-modeling results indicated that the hydrogen leak at GTU resulted in a maximum threat-zone distance of 4000 m for a flammable area of vapor cloud at night-time. Jet fire for the same plant with thermal radiation intensity of 37.5 kW/m^2^ reached a distance of 32 m and damaged nearby pipelines or vessels. Fireball radiations of thermal radiation intensity 37.5 kW/m^2^ for the leak of naphtha at CDU2 reached 83 m distance, and it will cause the spread of fire by domino effect to other areas. Jet fire thermal radiation of intensity 12.5 kW/m^2^ reached a maximum of 81 m from the source of fire for the naphtha leak at the CDU2 process plant. This intensity of the radiation may result in fire ignition. The flammable vapor clouds of hazardous leaks can cross the battery limits of the refinery and cause a threat to the neighboring society.

The suggested TRANCE computing model has been validated with high accuracy of 99.98% to predict the distance traveled by the chemical explosion of refinery incidents. The prediction results show that the coefficient of determination values in training, validation, and testing are 0.9998, 0.9998, and 0.9994, respectively. The mean absolute error values of the TRANCE model are 180.7024, 282.8242, and 679.5343. These highly accurate results are evident in the reliability of the proposed framework for safety distance prediction compared to the past results. Refinery management can adopt this model to assess the safety distance from the hazardous chemical explosion based on the prior forecasted-atmosphere conditions from the weather department. The proposed model of this research helps predict the threat zones and prepare the appropriate emergency response plan for the refinery. The results will also help plant designers plan the safety distances while siting the plants and selecting the construction material for the refinery plant and equipment to avoid a domino effect due to thermal radiation impingement. It will provide useful information to the regulatory authorities to prescribe mandatory safety distances, insurers, and disaster management authorities to prepare emergency plans.

## Figures and Tables

**Table 1:** The list of worst-case hazardous scenarios.

SNo.	Plant Location	Weather Condition	Situation	Leak Size	Pipe Dia	Pipe Length
1	GTU	5D	Catastrophic rupture of hydrogen pipeline in the summer daytime	FBR	300 mm	160 m
2	GTU	1.5F	Catastrophic rupture of hydrogen pipeline in the winter night-time	FBR	300 mm	160 m
3	CCR	5D	The leak of hydrogen pipeline in the summer daytime	150 mm	450 mm	137 m
4	CCR s	1.5 F	The leak of hydrogen pipeline in the winter night-time	150 mm	450 mm	137 m
5	GTU	5 D	The leak of gasoline pipeline in the summer daytime	150 mm	450 mm	250 m
6	GTU	1.5 F	The leak of gasoline pipeline in the winter night-time	150 mm	150 mm	250 m
7	KMU	5 D	Catastrophic rupture of kerosene pipeline in the summer daytime	FBR	450 mm	10 m
8	KMU	1.5 F	Catastrophic rupture of kerosene pipeline in the winter night-time	FBR	450 mm	10 m
9	MTBE	5 D	The leak of methanol pipeline in the summer daytime	150 mm	150 mm	30 m
10	MTBE	1.5 F	The leak of methanol pipeline in the winter night-time	150 mm	150 mm	30 m
11	CDU1	5 D	The leak of crude pipeline (IS2) in the summer daytime	FBR	450 mm	800 m
12	CDU1	1.5 F	The leak of crude pipeline (IS2) in the winter night-time	FBR	450 mm	800 m
13	CDU2	5D	The leak of naphtha pipeline (IS2) in the summer daytime	FBR	450 mm	30 m
14	CDU2	1.5F	The leak of naphtha pipeline (IS2) in the winter night-time	FBR	450 mm	30 m
15	ARU	5 D	The leak of toluene in the summer daytime	150 mm	150 mm	10 m
16	ARU	1.5 F	The leak of toluene in the winter night-time	150 mm	150 mm	10 m

**Table 2:** Overview of the TRANCE model.

Particulars	Specifications
Number of neurons in first and second hidden layer	5, 3
Number of features in input and output	3, 3
Training algorithm	Feed-forward back propagation
Optimization algorithm	Trainlm (Levenberg-Marquardt)
Activation function	Sigmoid and Linear
Performance-evaluation function	*MSE*, *R*^2^
Minimum number of epochs	15
Number of scenarios considered for risk assessment	163

**Table 3:** Performance of the developed TRANCE model for three zones.

	Observations	*MSE*	*R* ^2^
Training	113	180.7024	0.9998
Validation	24	282.8242	0.9998
Testing	24	679.5343	0.9994

## Data Availability

The data used in this work is incorporated in the paper itself.
